# An Improved IoT-Based System for Detecting the Number of People and Their Distribution in a Classroom †

**DOI:** 10.3390/s22207912

**Published:** 2022-10-18

**Authors:** Slavomir Matuska, Juraj Machaj, Robert Hudec, Patrik Kamencay

**Affiliations:** Faculty of Electrical Engineering and Information Technology, University of Zilina, 010 26 Zilina, Slovakia

**Keywords:** IoT-based system, IoT nodes, Raspberry Pi, Arduino-based module, COVID-19

## Abstract

This paper presents an improved IoT-based system designed to help teachers handle lessons in the classroom in line with COVID-19 restrictions. The system counts the number of people in the classroom as well as their distribution within the classroom. The proposed IoT system consists of three parts: a Gate node, IoT nodes, and server. The Gate node, installed at the door, can provide information about the number of persons entering or leaving the room using door crossing detection. The Arduino-based module NodeMCU was used as an IoT node and sets of ultrasonic distance sensors were used to obtain information about seat occupancy. The system server runs locally on a Raspberry Pi and the teacher can connect to it using a web application from the computer in the classroom or a smartphone. The teacher is able to set up and change the settings of the system through its GUI. A simple algorithm was designed to check the distance between occupied seats and evaluate the accordance with imposed restrictions. This system can provide high privacy, unlike camera-based systems.

## 1. Introduction

Following the COVID-19 outbreak in 2019, we have been facing different and difficult challenges in all aspects of our lives. One of them is without a doubt the continuous full-time educational process. Online education has its advantages, however, it cannot replace full-time education and student skills gained from face-to-face experience and practice, especially when it comes to education in technology and engineering. Various countries have different approaches that are enabling full-time education and access to facilities for students. Rules to reduce the maximum number of people in a classroom alongside social distancing rules have been introduced widely. These two mentioned restrictions were our primary motivation to propose an improved smart IoT-based system for detecting the number of people and their distribution in the indoor environment (e.g., classroom, or any type of room).

There have been a multitude of studies published in the area of detecting and counting people in indoor spaces. Most of the proposed solutions are based on image processing from cameras installed in the area. For example, Myint and Sein [1] proposed a robust camera-based system which is able to estimate the number of people entering and exiting a room. Their solution is based on Raspberry Pi and software using a pre-trained VGG-16 CNN model and an SVM classifier based on TensorFlow and the Keras library.

Another camera-based system for counting people using Raspberry Pi was proposed by Rantelobo et al. in [2]. This system can distinguish between people entering or leaving a room by performing image processing using background subtraction, morphological transformation, and calculating the contour area of the image. The main advantage of this solution is that the system can run on cheap hardware such as Raspberry Pi. Similar solutions relying on cameras and computer vision algorithms have been presented in [3,4].

Moreover, Hou et al. [5] presented a solution for social distancing detection based on a deep learning model. The primary goal was distance evaluation between individuals to mitigate the impact of the COVID-19 pandemic and reduce the virus transmission rate in indoor spaces. The detection tool was developed to alert people to maintain a safe distance from each other by processing a video feed from cameras used to monitor the environment. A similar system was proposed by Sharma in [6]. This system helps people to ensure proper social distancing in crowded places and highlights the violations of these norms in real time. The proposed system is based on image processing. There are many more published papers (e.g., [7,8]) solving the problem of social distancing using feeds from cameras and computer vision [9,10].

The work presented in [11] proposed a large Convolutional Neural Network (CNN) trained using a single-step model and You Only Look Once version 3 (YOLOv3) on Google Colaboratory to process the images within a database and accurately locate people within the images. The trained neural network was able to successfully generate test data, achieving a mean average precision of 78.3% and a final average loss of 0.6 while confidently detecting the people within the images. Yet another work presented in [12] uses YOLO v3 and Single Shot multi-box Detector (SSD) to detect and count people. The authors analyzed both methods and their comparison of the achieved results showed that the precision, recall, and F1 measure achieved for SSD were higher than for YOLO v3. The main issue related to camera-based systems involves privacy concerns, as data collected by cameras can be misused for face recognition, thus revealing the identity of the individuals in the area [13].

On the other hand, there are multiple works for counting people or measuring social distance which do not require the installation of camera systems. Among such systems, a smart social distancing monitoring system based on Bluetooth and GPS was described in [14]. In this system, an application can offer a solution for monitoring public spaces and reminding users to maintain distance. The work presented in [15] is based on an ultra-wideband radar sensor for a people counting algorithm. The proposed algorithm can operate in real time and is able to achieve a mean absolute error of less than one person. The system in [16] relies on Wi-Fi probing requests to count people in a crowd by taking advantage of people’s smartphones.

Another way of counting people or measuring social distance could be using Internet of Things (IoT) technology. The IoT can be described as a network of physical objects (things) that are equipped with sensors, software, and other technologies to connect and exchange data with other devices or systems via the internet or a local network. IoT represents interaction between the physical and the digital world in its simplest form. An IoT object in the world can be a simple sensor equipped with a communication interface or a smart self-driving car equipped with state-of-the-art technology. The advantages of using IoT technology in real-world applications are almost unlimited, and use cases can be found in such disparate areas as Industry 4.0 [17,18], smart agriculture [19,20], smart cities [21,22], smart transportation [23,24], smart homes [25,26], eHealth [27], and wearables [28]. With the massive adoption of IoT technology, it is finding applications in many areas [29,30]. For example, the authors of [29] stated that their platform, based on a combination of IoT and fog cloud, can be used in systematic and intelligent COVID-19 prevention and control. The system involves five use cases, including COVID-19 Symptom Diagnosis, Quarantine Monitoring, Contact Tracing and Social Distancing, COVID-19 Outbreak Forecasting, and SARS-CoV-2 Mutation Tracking [31]. Another IoT-based COVID-19 and Other Infectious Disease Contact Tracing Model was described by the authors of [32]. They presented an RFID-based proof-of-concept for their model and leveraged blockchain-based trust-oriented decentralization for on-chain data logging and retrieval.

The wearable proximity sensing system presented in [33] is based on an oscillating magnetic field that overcomes many of the weaknesses of the current state-of-the-art Bluetooth-based proximity detection. The authors proposed, implemented, and evaluated their system and demonstrated that the proposed magnetic field-based system is much more reliable than previously proposed Bluetooth-based approaches. Another possible solution for monitoring people in indoor environments was introduced by Perra et al. [34]. The proposed device implements a novel real-time pattern recognition algorithm for processing data sensed by a low-cost infrared (IR) array sensor. The device can perform local processing of infrared array sensor data, and in this way is able to monitor occupancy in any space of a building while maintaining people’s privacy. A seat-occupancy detection system based on Low-Cost mm-Wave Radar at 60 GHz was presented in [35]. Detection is based on Pulsed Coherent Radar in the unlicensed 60 GHz ISM band. The system can detect the presence of people occupying the seats by measuring small movements of the body, such as breathing. The solution for counting the people in the classroom proposed by Zhang et al. [36] is similar to the work presented in this paper. In their case, the authors used two E18-D80NK photoelectric sensors to count people in a classroom and an hc-sr501 infra-red sensor for detection of seat occupancy. However, the authors presented only the basic principles and hardware design of the system. The solution proposed in this paper is based on a slightly different technology with lower energy consumption. Moreover, it provides a complex solution with a user-friendly GUI and advanced functionalities, e.g., management of the rules and functions supporting deployment of the system.

The remainder of this paper is organized as follows. Section 2 is devoted to a description of the system concept. The system’s implementation is described in detail in Section 3, including the implemented methods, software, and hardware design. In Section 4, the achieved results are presented and discussed. Section 5 provides a comparison with other systems proposed for occupancy detection, and Section 6 concludes the paper.

## 2. System Concept

**Figure 1:**
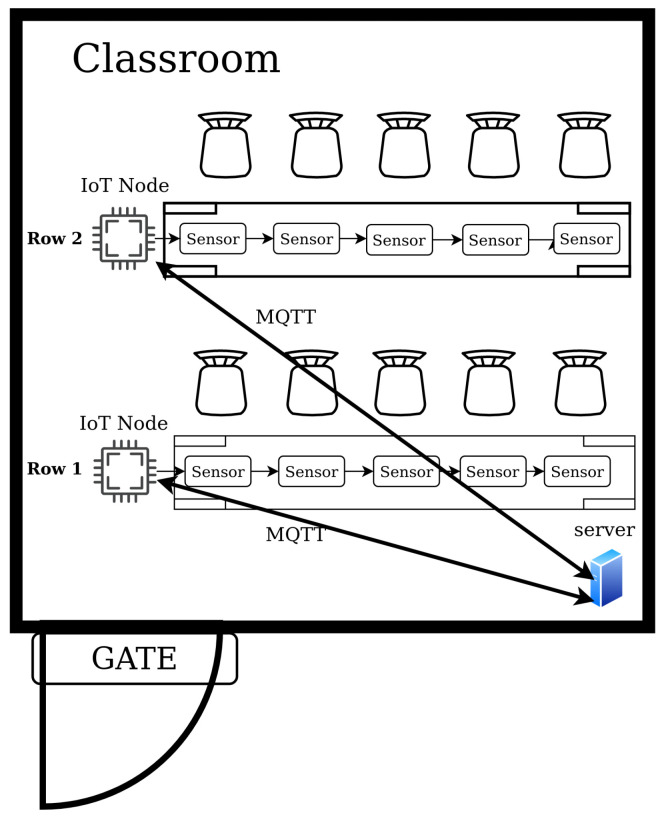
The system concept proposal.

The main goal of the proposed system is to deliver counting of incoming students when they are entering the classroom as well as detection of the distribution of the students among the seats. The proposed system is portable and can be easily deployed and managed by the teacher. The main purpose of the proposed system is to help teachers with the management of their classes while respecting implemented COVID-19 restrictions. The concept of the proposed system is presented in Figure 1.

The number of IoT nodes and sensor devices connected to the system is variable, however, there is one server per classroom. The teacher can access the system’s Graphical User Interface (GUI) from a mobile device or personal computer in order to set up the system as well as to check whether all restrictions are being obeyed. Each seat available in the classroom is equipped with a single HC-SR04 Ultrasonic Distance Sensor. Up to sixteen distance sensors can be connected to a single IoT node, which is responsible for the evaluation of the seat occupancy on a single row. Each IoT node collects data from the connected sensors and sends data via MQTT protocol to the main server. The IoT nodes are based on the NodeMCU Arduino board. The entrance to the classroom is equipped with a Gate node. The purpose of the Gate node is to count the students entering or leaving the classroom. The Gate node consists of a single NodeMCU board with two HC-SR04 sensors used for door crossing detection by persons entering or leaving the classroom. The HC-SR04 is a low-cost sensor which can provide distance measurements between 2 and 400 cm with non-contact measurements and ranging accuracy up to 3 mm. The sensor accuracy is sufficient for the purposes of occupancy detection. Each sensor module includes an ultrasonic transmitter, a receiver, and a control circuit. The sensor’s principle is as follows:

- To trigger measurement output, the pin has to be activated for at least 10 µs;
- The Module automatically sends eight 40 kHz signals and detects pulse signal reflections;
- The distance is calculated as follows:
  (1) $$\begin{array}{c} s_{t}=t*343/2, \end{array}$$
  where *t* is the pulse trigger duration and the constant 343 is the speed of sound in m/s.

The sensor implemented in the system operates at 5 volts and consumes 15 mA, while its dimensions are 45 ∗ 20 ∗ 15 mm. For the sake of comparison, the Infrared Proximity Sensor E18-D80NK used for people counting and HC-SR501 PIR sensor for detecting the seat occupancy in [36] consume 25–100 mA and 65 mA, respectively. Moreover, it is important to note that the infrared proximity sensor has a shorter sensing range compared to the ultrasonic sensor implemented in the proposed solution. On the other hand, the ultrasonic distance sensor has its own disadvantages, such as sensitivity to variations in the ambient temperature and difficulties when reading reflections from soft, curved, thin, and small objects. The server is based on the Raspberry Pi model 4B+ and is responsible for managing the whole system. The proposed solution consists of a Message Queuing Telemetry Transport (MQTT) broker for communication, Node-RED for logic, Mongo database for data storage, and a React-based web application that serves as the GUI. The system is deployed using Docker containers and the docker-compose tool ensures container orchestration.

### Cloud Technologies Versus Self-Hosted Solutions

There are two main categories of technology that can be implemented on the server side:

- Cloud-based technology;
- Self-hosted on-premises solutions.

Both of these have their advantages and disadvantages. Cloud technologies are relatively new platforms, however, they can offer a lot of already built-in features which are ready to use without a long setup. On the other hand, users may argue that when their data are not stored on their hardware, they do not have full control over the hardware, nor over the data. The primary goal of cloud technology for IoT is to provide universal functionality for application development as well as ubiquitous access to the data. Therefore, the user of the IoT platform can focus only on the functionality of its product and its value and does not need to care about the hardware itself. There are four categories of IoT platforms:

- IoT cloud platforms;
- IoT connectivity platforms;
- IoT device platforms;
- Analytics platforms.

The well-known IoT platforms in 2022 are represented by the IBM Watson IoT Platform, Particle, AWS IoT Core, Google Cloud IoT Core, Azure IoT Central, and many others. While many of the features provided by these platforms are free of charge, users must pay in order to receive the most out of each of these platforms.

The alternative is self-hosting. In this case, the user has to install and configure services according to their project’s needs. In the proposed system, a self-hosted solution based on the Raspberry Pi was chosen. The primary reason for this decision was that there may not be internet access available in every classroom and the Raspberry Pi can create its Wi-Fi network. Therefore, there is no need to add an extra router in order to provide connectivity for the system. With this in mind, the proposed system can be deployed in any classroom without any significant limitations.

## 3. System Implementation

**Figure 2:**
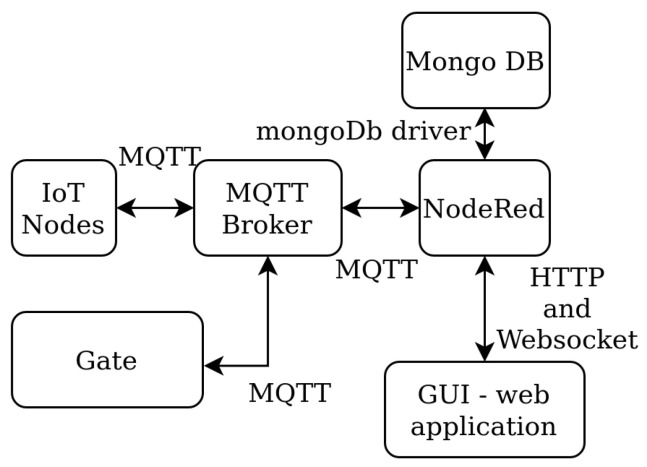
The main system data flowchart.

The first step in system implementation is the design of the communication flow diagram. The flowchart of the system functionalities is presented in Figure 2. Individual users can visit the GUI either from a personal computer in the classroom or through their smartphone. The communication between the GUI and the rest of the system was created using the HTTP protocol. WebSocket-based communication was implemented in order to obtain real-time information when seat occupancy changes. The main communication node of the system is the MQTT broker.

All communication between nodes and Node-RED applications is handled by this broker. All connected IoT nodes send their data to the broker. The Node-RED application is responsible for data processing and storing the results in the Mongo database server using the Mongo DB driver. At the same time, it provides a RESTful application programming interface (API) and WebSocket endpoint that serves the data for the GUI. The hardware of the system consists of three main blocks:

- Gate node;
- IoT nodes for detecting seat occupancy;
- Self-hosted server.

### 3.1. The Gate Node

**Figure 3:**
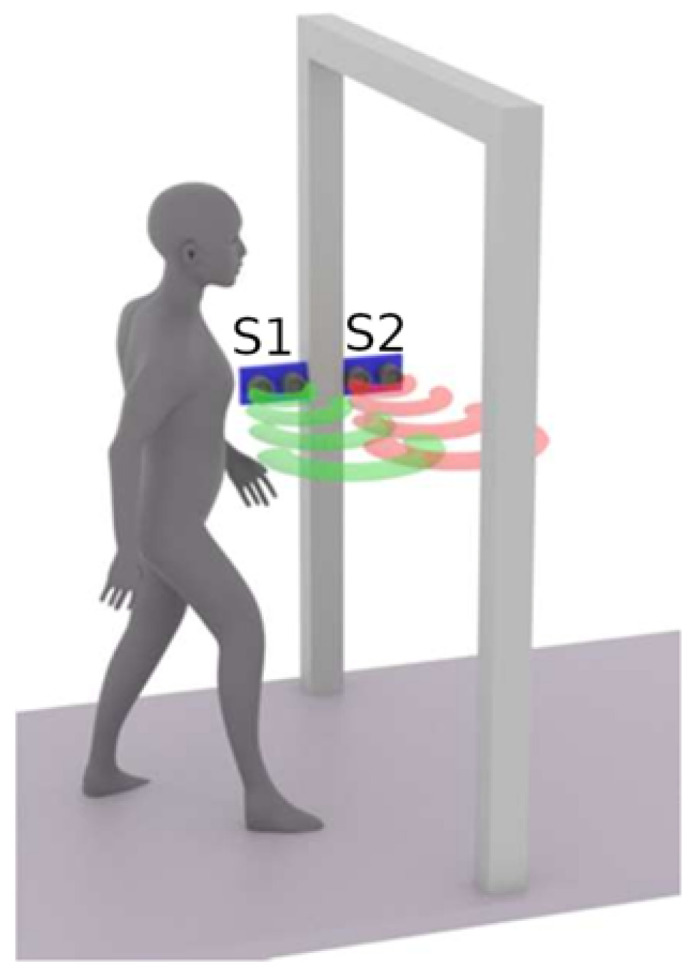
Principal functionality of Gate node.

The primary task of the Gate node is to detect people who cross the door, i.e., those entering or leaving the classroom. The Gate node consists of one NodeMCU board equipped with two HC-SR04 distance sensors. The principal functionality of the Gate node is depicted in Figure 3.

**Figure 4:**
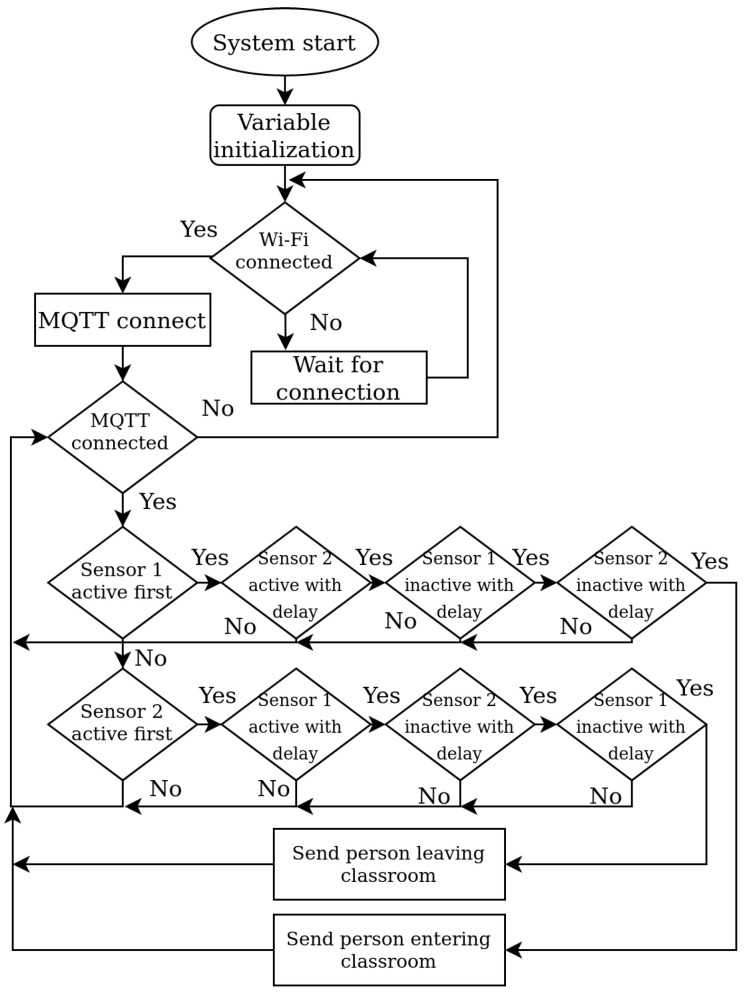
The Gate node algorithm implementation.

As mentioned earlier, from the sensing point of view the gate consists of two distance sensors, which are enough to detect when a person is crossing the door of the classroom. However, there is one limitation to this approach, being that only one person at a time can cross the door of the classroom. The flowchart of the software implemented in the Gate node is shown in Figure 4.

The software managing the Gate node starts with the initialization of variables and definitions. Moreover, it is necessary to set up the Wi-Fi network name (SSID), Wi-Fi password, server IP address, and MQTT credentials. When the node is connected to the Wi-Fi network as well as the MQTT broker, the Gate node starts to measure values from distance sensors. The PubSubClient library, which is available for Arduino-based boards, was used for connection and data transfer over MQTT. The MQTT topics used for this type of message are “sensors/gateEnter” and “sensors/gateExit”.

The algorithm for door-crossing detection operates based on the detection of a sequence of events reported by the sensors. To explain the crossing detection algorithm, we defined two states of sensors S1 and S2. The first is the “Active sensor”, which means that the measured distance from the sensor is shorter than the defined value of the door width, i.e., an active sensor means that the sensor detects the object. The second term is “Inactive sensor”, which means that the measured distance from the sensor is not shorter than the defined value of the door width, i.e., there is no object detected in front of the sensor. The possible sequences of sensor states resulting in successful crossing detection by the algorithm implemented in the Gate node are shown in Table 1, where 0 represents the inactive state of the sensor and 1 stands for the active state of the sensor.

In the table, steps t(0)–t(4) are defined by the time when the state of the sensor has changed. The system detects a door crossing event only when the states of the S1 and S2 sensors change according to the sequences provided in the table. In cases when other sequences are detected, the system does not detect a door crossing event, and thus does not change the number of persons in the room.

### 3.2. IoT Nodes for Detecting Seat Availability

**Figure 5:**
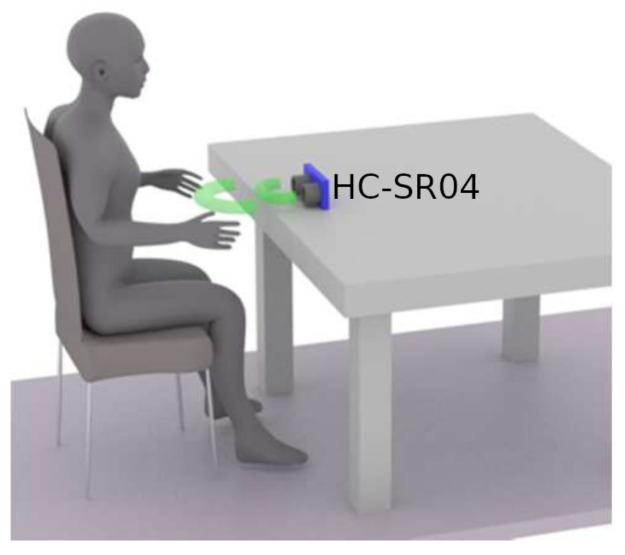
Sensor placement.

Each IoT node is based on a NodeMCU board which can connect up to sixteen distance sensors. In the proposed system design, it is necessary to use just a single distance sensor per seat. Therefore, it is possible to determine seat occupancy across the classroom and automatically check whether the students in the room are keeping the desired social distance simply by evaluating data from individual distance sensors. The ultrasonic distance sensor (HC-SR04) can be placed under the PC monitor or can be attached to the bottom part of the table. In order to connect sixteen distance sensors to the NodeMCU board, it is necessary to use a 16:1 single-channel analogue multiplexer, such as CD74HC4067 in our case. An example of the sensor placement is shown in Figure 5. The implemented sensor can provide distance measurements between 2 and 400 cm with non-contact measurements and ranging accuracy up to 3 mm. Each sensor module includes an ultrasonic transmitter, a receiver, and a control circuit. During operation, it is necessary to establish whether there is a person relatively close to the sensor. The decision-making distance was set at 70 cm during the tests, however, the teacher is able to change the decision-making distance based on the conditions in the classroom using the web application interface of the server. When the measured distance is shorter than the threshold value, the seat is considered to be taken, represented by a logical one; otherwise, the seat is considered to be free (logical zero).

**Figure 6:**
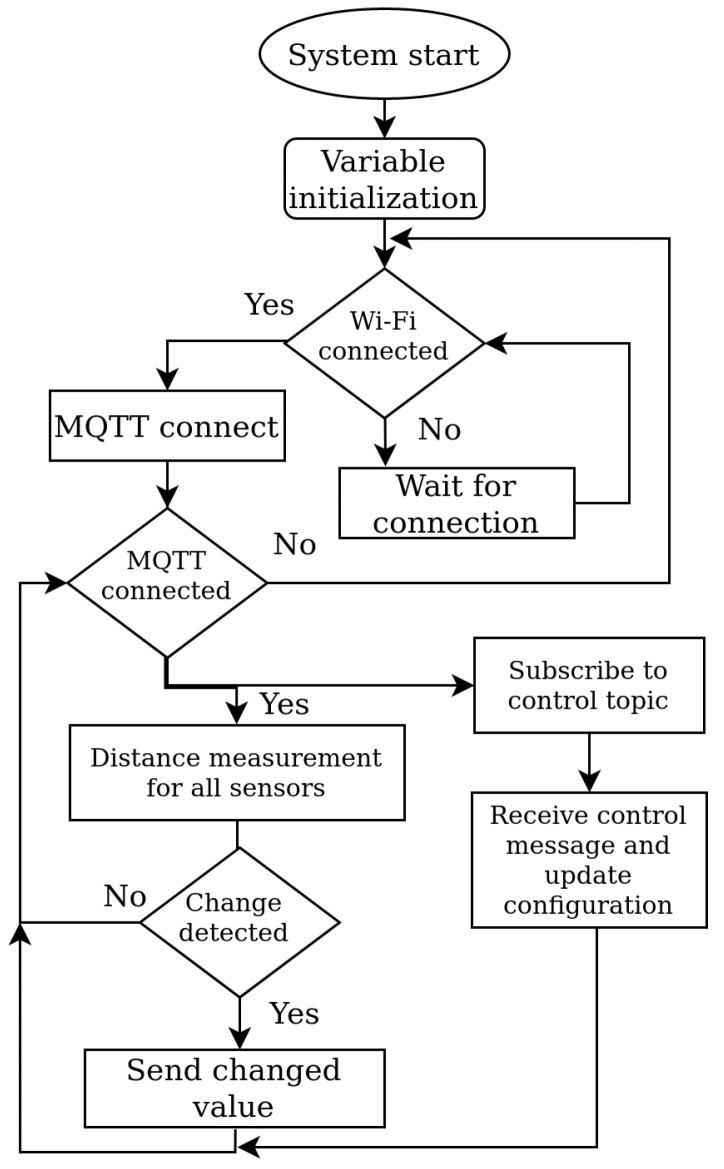
IoT node algorithm implementation.

When the seat occupancy status changes, the updated seat occupancy information is sent to the server for evaluation and storage. A flowchart of the software implementation for the IoT node for detecting seat availability is shown in Figure 6.

The software of the IoT node starts with variable initialization and definitions. The first part of the code matches the software for the Gate node, such as Wi-Fi and MQTT connections and initialization of libraries. Afterwards, the nodes measure the distances from each sensor in the loop. When a change in the sensor value is detected, the node sends the message to the server for evaluation. The MQTT topic used for this type of message is “/sensors/distanceChanged”. Useful information in messages represents JSON objects in string representation. A message with data about the occupancy sent from sensors looks like this:

{row: 1, seats: [0, 1, 0, 1, 0, 0, 0, 1]};

where the row specifies the position in the classroom and the seats represent an array of values that define the seat occupancy for individual positions in a row.

In the improved version of the IoT-based system, the option to set up the whole classroom from scratch using only the GUI was added. For this purpose, each node subscribes to the topic “nodes/setupConfig” to enable receiving of the configuration messages and is able send status data to the topic “nodes/nodeStatusUpdate”. The setup message for the IoT node is as follows:

{rowPosition: 1, sensorCount: 8, thresholdDistance: 70}; 

where rowPosition specifies the row position in the classroom, sensorCount defines the number of seats in a particular row, and thresholdDistance is used to set up the decision-making value for seat occupancy. The status message sent by the node contains the following information:

{nodeId: “011808db24be32c5”, nodeStatus: 0}; 

where nodeId is a string which represents the node’s unique identification in the system and NodeStatus defines the status of the node in the system. Node status can have two values, zero and one. The node sends a status message with nodeStatus equal to zero when it is connected to the system. The Node-RED application evaluates the message and checks whether the node is already registered in the system. In such a case, Node-RED responds with the configuration message to this node automatically. The node responds to Node-RED with the status message with the value of nodeStatus equal to one to confirm a successful setup. On the other hand, when the node ID is not registered in the system, i.e., a new node is connected, it needs to be configured from the GUI. The process of creating the classroom and configuring the nodes is described in Section 4.

### 3.3. Self-Hosted Server Solution

The server, which is the central unit of the proposed system, is based on the Raspberry Pi minicomputer. This device runs all applications and services that provide connectivity to IoT nodes as well as management of the system, data evaluation, and storage. There are five primary services running on the server: Mosquitto MQTT broker, Node-RED application, React web application, Nginx server, and MongoDB database system.

**Figure 7:**
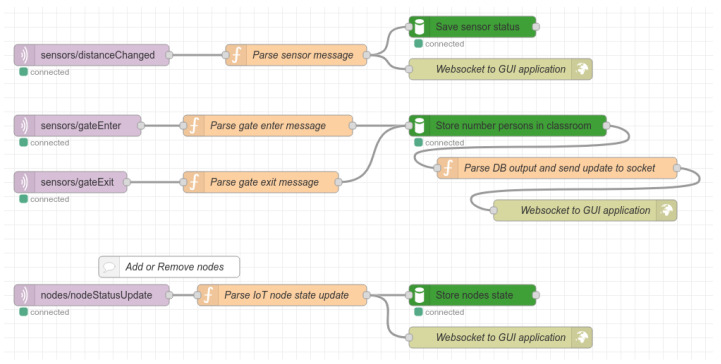
Schematic design of flow for handling incoming messages.

MongoDB is a popular general-purpose document-based distributed database that stores all data for actual and further evaluation. Mosquitto MQTT broker is an open-source MQTT message broker which is widely used across various IoT applications. The logic of the proposed system is implemented by a Node-RED application. The schematic design of flow for handling the messages from the Gate node for processing, evaluation, and representation of results is shown in Figure 7.

The flow starts with the MQTT broker input nodes. These input nodes listen to the topics “sensors/gateEnter” and “sensors/gateExit”. The purpose of this part of the flow is to collect data from the Gate node and evaluate the number of students that are currently in the classroom. All pieces of information are stored in the Mongo DB, and thus are available to Node-RED. However, the latest values are stored in flow variables as well, and their updates are sent via WebSocket to the connected client using the React web application. The message with the number of people currently in the classroom appears as follows:

{“counter”:14, “limit”:20}; 

where the counter represents the number of people and the limit is the maximum allowed amount of people who can be inside the room due to active restrictions. This limit could be changed by a teacher via the GUI.

Another MQTT broker input node listens to the topic “/sensors/distanceChanged”. This node is responsible for handling the incoming messages from the IoT nodes about changes in seat occupancy. The structure of this message was provided in the previous section.

**Figure 8:**
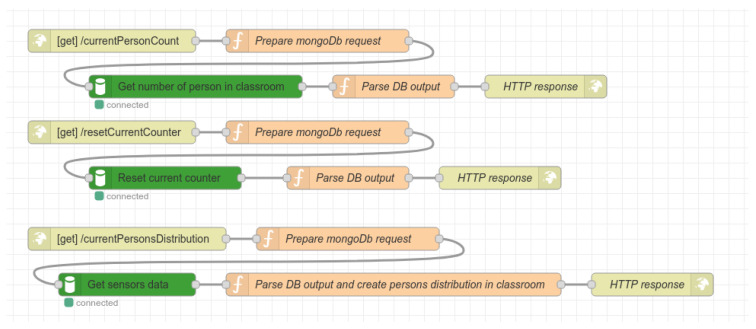
Schematic design of flow for providing the RESTful API.

The next part of the flow is responsible for providing all system data as the RESTful API. An example of this flow design is depicted in Figure 8. Altogether, there are eight different routes implemented in the API; three are POST routes, while the rest are GET routes. The description of the RestAPI GET routes is as follows:

- /currentPersonCount: the route is responsible for obtaining information about the actual number of persons in the classroom. The route flow acquires the data from the database and creates a response in JSON format;
- /resetConterCount: the route for resetting the current counter. For example, this can be used in cases when false detection occurs;
- /currentPersonsDistribution: the route obtains the data from the database about actual person distribution in the classroom. Data are then stored in JSON format and sent to the client. The data structure is described later in the experimental results section;
- /getDevicesList: the route for acquiring the actual list of all IoT nodes connected to the system. Data are acquired from the database as well;
- /getLessonsList: the route obtains all lessons records from database and returns them as JSON objects.

Description of RestAPI POST routes:

- /setupDevice: this route serves to set up the IoT node. It expects the JSON data in the request body with parameters specifying node position, sensor count, and threshold for decision-making distance when the seat is considered occupied;
- /startLesson: this route starts a new lesson; it expects only one parameter, e.g., lesson name;
- /finishLesson: this route finishes the current lesson, and is parameter-less.

**Figure 9:**
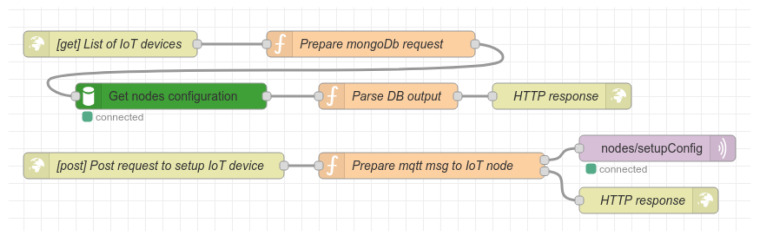
Schematic design of flow for providing the RESTful API for setting up the IoT nodes.

Two of the routes shown in Figure 9 handle the IoT nodes’ configuration setup from the GUI. The configuration process of the IoT nodes is described in the next section.

Simple algorithms were designed to evaluate whether the distribution of students across the classroom meets the requirements defined by COVID-19 restrictions. It is assumed that the students are seated in rows; however, there do not have to be the same number of seats in each row. The implemented algorithm considers student distribution to be valid when the distance between taken seats is at least 2 in both the x and y directions. Otherwise, the system shows a popup notification about the violation of the restriction.

## 4. Experimental Results

**Figure 10:**
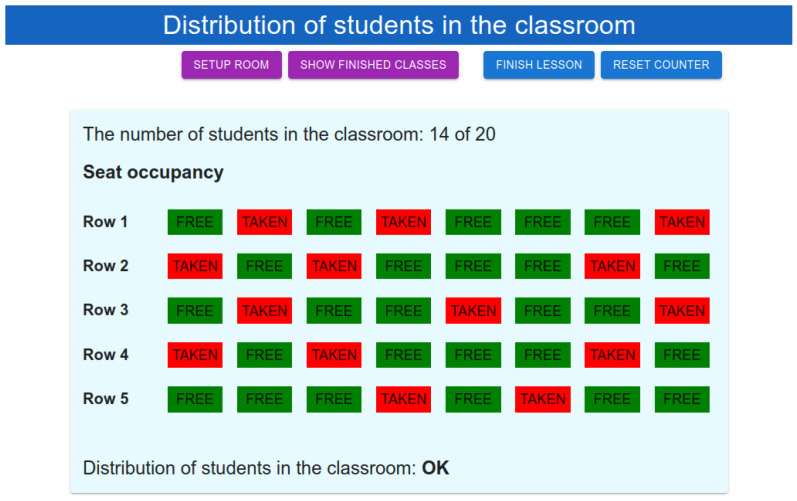
GUI home screen showing student distribution in the classroom.

In this section, the GUI of the proposed IoT-based system for detecting the number of people and their distribution in the classroom is presented. The application helps the teacher to easily handle the implementation of restrictions defined due to the COVID-19 outbreak. The home screen of the developed web application is shown in Figure 10.

The teacher’s daily routine will be as follows. Before the beginning of the class, the teacher enters the classroom and resets the current count of the students in the classroom. Afterwards, students can enter the classroom. The web application shows any changes in seat occupancy in real time using WebSocket communication. When students are heading to their chosen seat, they can cross other seats and temporarily change seat occupancy status. This could lead to an evaluation of person distribution that does not meet the requirements defined by COVID-19 restrictions. The system shows an alert only when the incorrect seat occupancy state holds for more than one minute. In a typical scenario, the system evaluates the data in real-time, and therefore temporarily changing seat status occupancy does not cause an alert. All other communication between the web application and the Node-RED backend is carried out via HTTP protocol. The data received from WebSocket or an HTTP GET request representing the student distribution are defined as follows: 

{

distributionState: “Ok”,

data: [

  {row: 1, seats: [0, 1, 0, 1, …, 0, 1]};

  {row: 2, seats: [1, 0, 0, 0, …, 1, 0]};

  …

  {row: n, seats: [0, 1, 0, 1, …, 0, 1]};

]} 

where “distributionState” tells whether the students’ distribution across the classroom meets the requirements defined by restrictions. The entry “data” represents the real seat occupancy in the classroom. When the teacher finishes the lesson, all information gathered during the lesson is stored in the database for later analysis. The teacher can check the relevant data from the finished lessons at any time.

**Figure 11:**
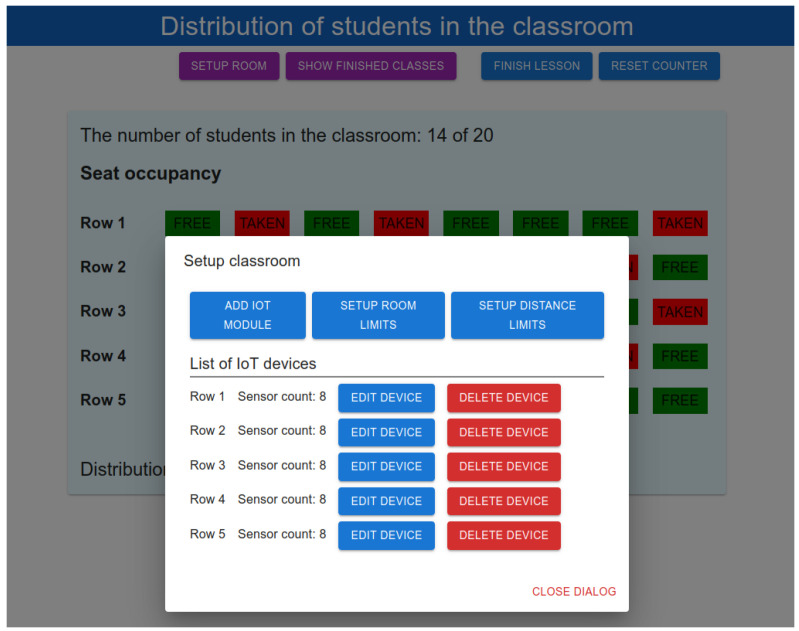
GUI for classroom setup.

The user window that provides the classroom setup and configuration is shown in Figure 11. The proposed system was designed to be as simple as possible from both the setup and implementation point of view. After all software is installed on the Raspberry Pi, the user is able to configure the classroom via the GUI. This configuration process is as follows. First, the user needs to set up the room limits, such as the maximum number of students in the classroom and minimum distance limits. Afterwards, the teacher turns on the first IoT node in the first row. The IoT node is registered in the system. By clicking on the button “Add IoT module”, the teacher can display a window with a list of all unconfigured nodes in the system. Then, the teacher selects one of the unconfigured IoT nodes and configures it by setting the position in the classroom (e.g., the row number parameter) and the number of sensors connected to the IoT node, which is the number of seats in a particular row. Then, the IoT node is ready for use, and the teacher can add the rest of the IoT nodes. Moreover, the teacher is able to change the configuration of any IoT node at any time.

Experiments were carried out to test the robustness and reliability of the door crossing detection algorithm at the Gate node. The tests were performed considering the following scenarios:

1. The person enters the classroom.
2. The person leaves the classroom.
3. The person enters the Gate area, stops, and then continues in the same direction.
4. The person enters the Gate area, stops, and leaves in the direction from which they entered.

Each scenario was tested 100 times, and the achieved results are presented in Table 2.

From the achieved results, it is obvious that the proposed system is both robust, and reliable and is able to correctly detect door crossing using the Gate node. The Gate node is able to distinguish between all four cases, i.e., a person who enters the room, leaves the room, or decides to return after entering the door from either side. The Gate can detect other crossing objects that are not human targets, such as bags, cabinets, or tables. However, it is not possible to distinguish what type of object crosses through the Gate. Moreover, the system can operate in real time and is designed to be deployed at a relatively low cost. The hardware cost of equipment required for one classroom with a capacity of 40 seats, i.e., five rows and eight seats per row, is approximately EUR 210, as can be seen in Table 3.

It is important to note here that deployment of the system does not require any preexisting infrastructure for the internet connection. The server, running on Raspberry Pi, can provide wireless connection to all IoT nodes in the room and store all the data locally.

## 5. Discussion

In this section, the proposed solution for counting and detecting the distribution of people around the classroom is compared with the other state-of-the-art works. As our literature review found only a single work dealing with a similar solution based on data from a sensor network, solutions based on image processing were considered in comparison with proposed system. It is important to note that in most papers a limited amount of information about the systems requirements, cost, and power consumption were presented. However, based on the provided information, several parameters for comparison could be estimated. For the comparison, implementation of the system for the classroom with a capacity of 40 seats, i.e., five rows and eight seats per row, was considered. Unfortunately, due to the lack of information provided in the literature, it is not possible to cover all comparison parameters for all solutions. The comparison of the proposed system with other solutions is shown in Table 4.

From the comparison, it is clear that the proposed system can provide information about seat occupancy with high accuracy while maintaining the privacy of people in the monitored area. In addition to the privacy, the advantage of the proposed system over systems based on image processing is that accuracy is consistent over whole area, while in systems based on image processing the accuracy can be affected by the position of the camera. On top of that, image processing-based solutions are not designed to provide information about distribution of people in the area. Moreover, the proposed system can operate in real time with low implementation cost and lower power consumption than the IoT-based solution proposed in [36].

## 6. Conclusions

In the paper, an improved IoT-based system for detecting the number of people in a classroom and their distribution was proposed. The main purpose of the proposed system is to help teachers to manage their classes with respect to rules implemented due to COVID-19 restrictions. An improved system was presented in which the teacher can set up the whole classroom from a GUI. The system is more robust and much easier to extend than the previous version published in [37]. The teacher is able to configure IoT nodes from the GUI and change the configuration at any time. The system consists of a Gate node for counting people entering or leaving the classroom, IoT nodes with distance sensors placed in the room for detecting the availability of seats, and a server based on a Raspberry Pi. It is possible to connect up to sixteen HC-SR04 ultrasonic distance sensors to a single IoT node; thus, a single node is able to check the availability of sixteen seats. The Raspberry Pi server can create a Wi-Fi network, which is used to transfer the data from the IoT nodes; therefore, there is no need for an extra Wi-Fi router to provide connectivity in the classroom. Five system services running on the Raspberry Pi provide all functionalities of the proposed system, which uses an Nginx server for request routing, a React web application, the Mongo DB database, a Node-RED application for the logic part, and an eclipse-mosquitto MQTT broker. All services run on the server as Docker containers. Thanks to this setup, it is easy to deploy the proposed system in any classroom. The main idea of our system is to help teachers to handle COVID-19 restrictions. However, there may be other use cases for the system. For example, the system can check the distance between students during exams and tests in order to help prevent cheating. The main disadvantage of the proposed system is the complexity of the deployment of the IoT modules compared to camera-based systems. When camera-based systems are deployed, it is sufficient to simply place the camera in the classroom and the system is ready immediately. However, in the proposed system it is necessary to connect cables between sensors and IoT nodes in order to provide the power supply for the individual IoT nodes. On the other hand, when privacy is considered, the proposed system has the advantage in that it cannot provide any information about the identities of people in the room.

The proposed system was deployed and tested in a classroom at the University of Zilina. The presented IoT-based system is highly reliable and robust thanks to the algorithm’s simplicity and clarity. The main advantage of the system is the possibility of deployment without jeopardizing the privacy of individuals in the classroom, as there is no way to identify individuals, unlike in camera-based systems.

## Figures and Tables

**Table 1:** Sequences for successful door crossing detection.

Result	t(0)		t(1)		t(2)		t(3)		t(4)	
	S1	S2	S1	S2	S1	S2	S1	S2	S1	S2
Person entered room	0	0	1	0	1	1	0	1	0	0
Person left room	0	0	0	1	1	1	1	0	0	0

**Table 2:** Gate node implementation testing results.

Scenario	Number of Tests	Correct Detection	Incorrect Detection	Accuracy [%]
1.	100	100	0	100
2.	100	100	0	100
3.	100	100	0	100
4.	100	100	0	100

**Table 3:** Cost of system deployment.

Item	Unit Price	Count	Total Price [Eur]
Raspberry PI	40	1	40
NodeMCU	5	6	30
HC-SR04	1.4	42	58.8
74HC4067 (16-channel multiplexer)	2.3	5	11.5
Wires, pcb and secondary materials	70	1	70
Total			210.3

**Table 4:** Comparison of the proposed system with other solutions.

Solution	Proposed Solution	Zhang et al. [36]	Le et al. [4]	Hasan et al. [11]	Al-Sa’d et al. [7]
Initial investment [Eur]	210	220	80	330	minimal estimated price: 330
Power consumption [W]	8.55	14.5	4.425	300 (Tesla K80 + camera)	minimal estimated: 300
Privacy	respected	respected	not respected	not respected	not respected
Robustness against physical tempering	part of the system could be easily damaged	part of the system could be easily damaged	depends on the camera position	depends on the camera position	depends on the camera position
System accuracy	Very high, easy and precise algorithms	not applicable	not provided	78.3% (mAP)	F1—99.1
Provides distribution of people	yes	yes-not in GUI	no	Possible to calculate, but not provided	Possible to calculate, but not provided
Response time	Real-time	Real-time	7.5 fps	not provided	Real-time

## Data Availability

The data are available from the authors upon request.

## Abbreviations

The following abbreviations are used in this manuscript:

GUI	Graphical User Interface
HTTP	Hypertext Transfer Protocol
ID	Identification
IoT	Internet of Things
MQTT	Message Queuing Telemetry Transport
